# Study of cochlear microphonic potentials in auditory neuropathy^☆^

**DOI:** 10.1016/j.bjorl.2015.11.022

**Published:** 2016-04-27

**Authors:** Ilka do Amaral Soares, Pedro de Lemos Menezes, Aline Tenório Lins Carnaúba, Kelly Cristina Lira de Andrade, Otávio Gomes Lins

**Affiliations:** a Universidade Federal de São Paulo (UNIFESP), Ciências Médicas, São Paulo, SP, Brazil; b Universidade Estadual de Ciências da Saúde de Alagoas, Maceió, AL, Brazil; c Universidade de São Paulo (USP), São Paulo, SP, Brazil; d Universidade Federal de São Paulo (UNIFESP), São Paulo, SP, Brazil; e Universidade Federal de Pernambuco (UFPE), Recife, PE, Brazil

**Keywords:** Cochlear microphonic, Cochlear microphonic potential, Hearing loss, Microfonismo coclear, Potencial microfônico coclear, Perda auditiva

## Abstract

**Introduction:**

Auditory Neuropathy/Dyssynchrony is a disorder characterized by the presence of Otoacoustic Emissions and Cochlear Microphonic Potentials, an absence or severe alteration of Brainstem Evoked Auditory Potential, auditory thresholds incompatible with speech thresholds and altered acoustic reflexes. The study of the Cochlear Microphonic Potential appears to be the most important tool for an accurate diagnosis of this pathology.

**Objective:**

Determine the characteristics of the Cochlear Microphonic in Auditory Neuropathy/Dyssynchrony using an integrative review.

**Methods:**

Bibliographic survey of Pubmed and Bireme platforms and MedLine, LILACS and SciELO data banks, with standardized searches up to July 2014, using keywords. Criteria were established for the selection and assessment of the scientific studies surveyed, considering the following aspects: author, year/place, degree of recommendation/level of scientific evidence, objective, sample, age range, mean age, tests, results and conclusion.

**Results:**

Of the 1959 articles found, 1914 were excluded for the title, 20 for the abstract, 9 for the text of the article, 2 for being repeated and 14 were selected for the study.

**Conclusion:**

The presence of the Cochlear Microphonic is a determining finding in the differential diagnosis of Auditory Neuropathy/Dyssynchrony. The protocol for the determination of Cochlear Microphonic must include the use of insert earphones, reverse polarity and blocking the stimulus tube to eliminate electrical artifact interference. The amplitude of the Cochlear Microphonic in Auditory Neuropathy/Dyssynchrony shows no significant difference from that of normal individuals. The duration of the Cochlear Microphonic is longer in individuals with Auditory Neuropathy/Dyssynchrony.

## Resumo

**Introdução:**

A Neuropatia/Dessincronia Auditiva é uma doença caracterizada pela presença das Emissões Otoacústicas e do Microfonismo Coclear, com ausência ou grave alteração do Potencial Evocado Auditivo de Tronco Encefálico, limiares auditivos incompatíveis com limiares vocais e reflexos acústicos alterados. O estudo do Microfonismo Coclear parece ser a ferramenta mais importante para um diagnóstico preciso desta patologia.

**Objetivo:**

Verificar por meio de uma revisão integrativa as características do Microfonismo Coclear na Neuropatia/Dessincronia Auditiva.

**Método:**

Levantamento bibliográfico nas plataformas Pubmed e Bireme e nas bases de dados MedLine, LILACS e SciELO, com buscas padronizadas até julho de 2014, utilizando-se palavras-chave. Para a seleção e avaliação dos estudos científicos levantados, foram estabelecidos critérios, contemplando os aspectos: autor, ano/local, grau de recomendação/nível de evidência científica, objetivo, amostra, faixa etária, média de idade em anos, testes, resultados e conclusão.

**Resultados:**

Dos 1959 artigos encontrados, 1914 foram excluídos pelo título, 20 pelo resumo, nove pela leitura do artigo, dois eram repetidos e 14 foram selecionados para o estudo.

**Conclusão:**

A presença do Microfonismo Coclear é um achado determinante no diagnóstico diferencial da Neuropatia/Dessincronia auditiva. O protocolo de registro do Microfonismo Coclear deve contar com o uso de fones de inserção, a inversão da polaridade e o bloqueio do tubo do estímulo para impedir a interferência de artefato elétrico. A amplitude do Microfonismo Coclear na Neuropatia/Dessincronia auditiva não apresenta diferença significante entre a amplitude do Microfonismo Coclear em ouvintes normais. A duração do Microfonismo Coclear é maior em indivíduos com Neuropatia/Dessincronia auditiva.

## Introduction

The term auditory neuropathy (AN) was first used in 1996 to define a group of individuals with auditory symptoms, who had in common normal cochlear function despite having abnormal cochlear nerve function. Moreover, they experienced difficulty in understanding speech especially in noisy environments, although in some cases they responded to sound stimuli.^1^ Today the most common denomination is auditory neuropathy/dyssynchrony (AN/AD).

In general findings reveal the absence or severe abnormality of the Auditory Brainstem Response (ABR) with preservation of the otoacoustic emissions (OAE) and/or the Cochlear Microphonic (CM), indicating disordered function of the auditory nerve with normal function of the cochlear hair cells (HC).^1^, ^2^, ^3^, ^4^

It is often difficult to determine exactly the onset of AN/AD, but the disease can occur at all ages.^4^ Its prevalence has been estimated at 11% in a group of 109 hearing-impaired children who failed the newborn hearing screening (NHS) and ABR.^5^ Another study reports a similar prevalence of 8.44% in 379 children evaluated with ABR alteration.^4^

The CM is a potential generated from the outer hair cells (OHC) and inner hair cells (IHC) of the cochlea and its absence is consistent with alterations in the function of these cells.^2^, ^6^ It is an electrical activity that precedes the synapses of the HC with the auditory nerve and, therefore, when recorded, it appears before wave I on ABR and maintains its latency even when the stimulus intensity is decreased.^5^

There are still no available data regarding CM parameters in individuals with normal hearing or with hearing loss. However, recording the CM attracted renewed interest after the identification of the AN/AD,^1^ as the association between the cochlea and an acoustic stimulation has been used in the differential diagnosis of AN/AD, once the presence of CM can be used as evidence of OHC integrity.^7^

The literature recommends that tests of cochlear function, particularly CM, become part of the NHS (Newborn Hearing Screening) protocol in all children with absent or altered ABR, facilitating the diagnosis of AN/AD.^5^

The aim of the study is to verify the characteristics of cochlear microphonism in Auditory Neuropathy/Dyssynchrony through an integrative review.

## Methods

The methodological process characterized the present study as an integrative review, to gather data from studies that help the understanding of the subject in a systematic and orderly manner, thus helping to acquire further knowledge on Cochlear Microphonic characteristics in Auditory Neuropathy/Dyssynchrony.

The integrative review was carried out from electronic searches in Pubmed and Bireme platforms and in the following databases: MedLine, LILACS and SciELO – Regional. The data search was started and concluded in July 2014. Studies published in English, Spanish or Portuguese were selected for the analysis. There was no restriction regarding the year of publication, i.e. studies published up to July 2014 were analyzed, and subsequently, the articles were selected according to inclusion and exclusion criteria.

The search strategy was performed by crossing the descriptors (DeCS and MeSH), as well as the free terms, which are terms not found in MeSH and MeSH, but that are relevant to the search. The descriptors used to locate the studies were Cochlear Microphonic and Cochlear Microphonic Potential and the free terms used were Auditory Neuropathy and Auditory Dyssynchrony.

### Search strategy

The search strategy was directed by a specific question: “What are the characteristics of the Cochlear Microphonic in Auditory Neuropathy/Dyssynchrony?”. Aiming to identify the relevant articles with the proposed question, a search strategy was developed, using the descriptors in groups, with at least two keywords. The descriptors used were: *Cochlear Microphonic/Auditory Neuropathy/Auditory Dyssynchrony/Cochlear Microphonic* AND *Auditory Neuropathy* OR *Auditory Dyssynchrony/Cochlear Microphonic Potential/Auditory Neuropathy/Auditory Dyssynchrony/Cochlear Microphonic Potential* AND *Auditory Neuropathy* OR *Auditory Dyssynchrony.*

### Selection criteria

#### Inclusion criteria

Articles with the following characteristics were included: original article, case report or literature review including as research subjects individuals diagnosed with auditory neuropathy.

#### Exclusion criteria

The articles that did not describe the findings of audiological assessment in individuals with AN/AD were excluded from this review.

### Study identification, selection and inclusion

The study was independently carried out by two researchers and the points of conflict were discussed at specific meetings. After applying the search strategy containing the defined descriptors, article selection was performed in three stages:

1. Identification and reading of titles in different electronic databases. Articles that clearly did not meet any of the inclusion criteria of this study were excluded.
2. Reading of summaries of the studies selected at the first stage. Similarly, we excluded articles that clearly did not meet any of the pre-established inclusion criteria.
3. All studies that were not excluded in these first two stages were read in full for the selection of those that would be included in this review.

All studies used met the inclusion criteria defined in the beginning of the methodological protocol of this study, aiming to answer the question that guided this integrative review. The main data of each article were fully collected and entered into a Microsoft Office Excel 2011 program database.

For better presentation of the results, it was decided to consider the following variables of the selected articles: author, year/location, type of study, grade of recommendation/level of scientific evidence, objective, sample, age range, mean age in years, tests performed, results and conclusion.

As for the level of scientific evidence, the classification used was that of Oxford Center for Evidence-Based Medicine.^8^

## Results

According to the performed search, 1959 articles were found in the electronic searches. According to the inclusion and exclusion criteria defined in the method and after eliminating the repeated references found in more than one database, 14 articles were selected.

In the MedLine database, via PubMed, after employing the keywords and free terms, 1959 articles were found, of which 1913 were excluded after reading the title, 44 abstracts were read and 25 articles were selected for reading in full. Of these 25, two were repeated articles and nine were excluded. In the LILACS and MEDLINE databases via Bireme, no articles were found for this search. Two articles were found in the SciELO database; one was excluded after reading the title and the other was excluded after being read in full.

**Figure 1:**
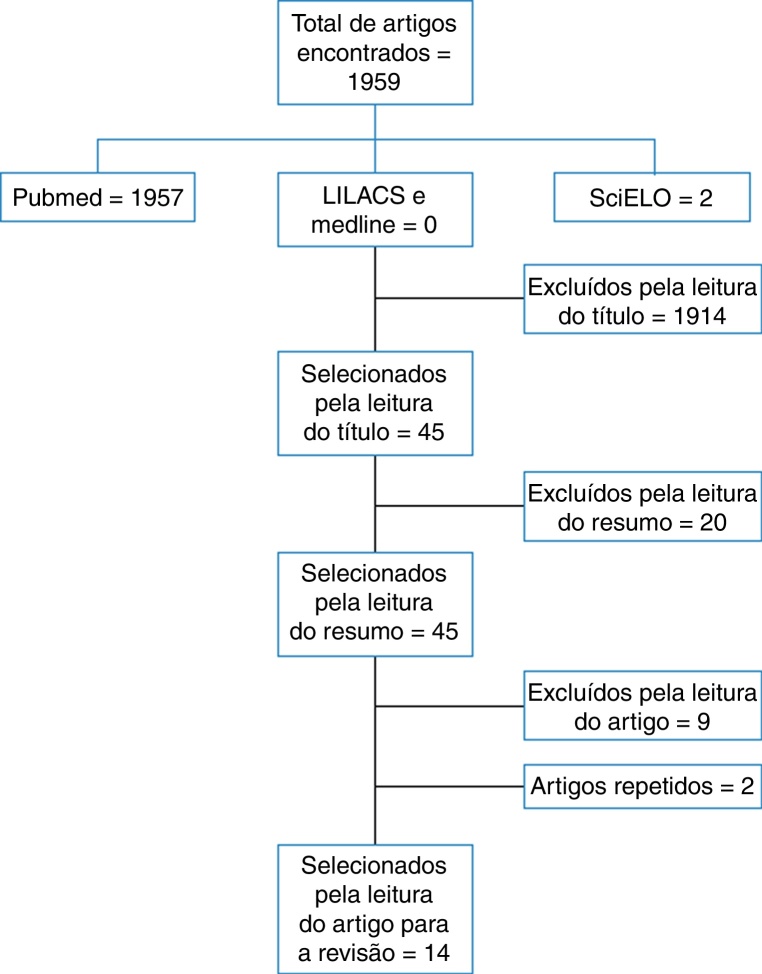
Flowchart of articles identified, excluded and included in the integrative review.

The following flow chart (Fig. 1) is a synthesis of the article selection process for the integrative review.

Table 1 is a synthesis with the characteristics of the studies included in the integrative review.

**Table 1:** Summary with characteristics of the studies included in the review.

Author	Year/place	Study type	Grade of recommendation/level of scientific evidence	Objective	Sample	Are range in years	Mean age in years	Tests	Results	Conclusion
Deltenre et al.	1997/Belgium	Case study	C/4	Describe a new form of hearing dysfunction characterized by absent ABR, with evidence of function of the outer hair cells of the cochlea, the cochlear microphonic potential and preserved OAE	3	0–4 months	Not reported	ABR (clipped), OAE, acoustic immittance testing	OAE present, absent ABR with the presence of CM, residual hearing in one case in Behavioral Audiometry, normal tympanogram and contralateral acoustic reflexes present	OAE and ABR alone may indicate an unusual situation, but the verification only occurs with the recording of CM. Recognition of the microphonic potential isolated from routine recordings facilitated by the use of reverse polarity can be valuable for the neurophysiological evaluation of peripheral hearing and thus, it is highly recommended.
Santarelli and Arslan	2002/Italy	Case study	C/4	Describe the findings of the ECoG in 5 patients, one adult and four children, with absent ABR and presence of DPOAE	5	3 months to 19 years	Not reported	EcogT, ABR click DPOAEs, acoustic immittance testing, Behavioral Audiometry, Vocal Audiometry	In some cases EcogT was the only reliable diagnostic tool to detect peripheral damage such as brainstem generator dysfunction	EcogT in AN provides a reliable assessment of peripheral auditory function, allowing some hypotheses about the lesion site
Rapin and Gravel	2003/USA	Literature review	D5	Identify an adequate term for diseases that affect the central auditory pathway in the brainstem and, selectively, in the brain	Not applicable	Not applicable	Not applicable	Not applicable	Pure auditory neuropathy is rare, in many cases, both the 8th nerve and central auditory pathway or, in some cases, CC contribute to atypical hearing loss and speech recognition	The term AN is not adequate for cases in which the pathology is predominantly in the brainstem and should be reserved for patients with evidence that the disease involves the spiral ganglion cells and their axons
Berlin et al.	2003/USA	Literature review	D5	Study of AN diagnosis and management	Not applicable	Not applicable	Not applicable	Not applicable	Studies performed in the last 20 years show that although the electroacoustic evaluation can provide good diagnosis, these responses are products of a complex physiological process and are not necessarily the true perception indicators	The attempts to characterize several aspects of the AN profile have shown that the results demonstrate a common physiological pattern due to different pathological processes, or different degrees of involvement
Rance	2005/Australia	Literature review	D5	Studying the mechanisms of AN, type of disorder, clinical profile of patients and mainly the effects of the perception of AN, which are quite different from those associated with SNHL	Not applicable	Not applicable	Not applicable	Otoscopy EcogT, ABR click, DPOAEs, acoustic immittance testing	The results show that in all patients, amplitude and CM threshold are critically dependent on the CAP threshold, showing an association of CM with both OHC and IHC	The presence of a CNS disorder seems to improve the CM amplitude. In some cases, the disappearance over time of DPOAE suggests that changes in the amplitude and duration of CM in patients with AN, result from a combination of loss of OHC and alterations in the efferent system
Santarelli et al.	2006/Italy	Observational Cross-sectional	C4	Evaluate the amplitude of the CM and the hearing threshold of normal ears and ears with varying degrees of elevation in the recording of the Action Potential and compare with the corresponding values in a group of patients with AN	522	7 months to 47 years	3.1 years	Pure tone and vocal audiometry, acoustic immittance testing, OAE and ABR	The CM amplitude was significantly higher in patients with CNS disease than in those with normal hearing. CM responses were detected in all auditory patients with AN, with amplitudes and thresholds similar to those calculated for individuals with normal hearing. The duration of the CM was significantly higher in the group with AN	1. CM detection is not a distinctive characteristic of AN; 2. Patients with CNS disease showed an increase in amplitude and duration of CM, possibly due to the efferent system dysfunction; The duration, high frequency and amplitude of the CM were similar in patients with normal hearing and AN. This may result from a variable combination of the type of efferent system lesion and loss of OHC
Anastasio et al.	2008/Brazil	Case report	D5	Demonstrate the clinical applicability of EcogET in the differential diagnosis of AN when compared to ABR	1	4 years	Not applicable	OAE, ABR click, ABR 0.5 and 1 kHz tone pips and imaging test	A 4-year-old child, diagnosed with AN underwent the Ecog-ET with 2000 Hz tone burst in rarefaction and condensation polarities	Ecog-ET allowed a more detailed analysis of CM compared to the ABR, thus showing clinical applicability for the investigation of cochlear function in AN
Ahmmed et al.	2008/United Kingdom	Case report	C4	Study the diagnostic dilemma about the presence of CM together with a significant increase in ABR thresholds in infants who fail at NHS	1	6 weeks	Not applicable	TOAE, Ecog, ABR by click, by BC and Toneburst (500, 1000 and 2000 Hz)	SNHL diagnosed through clinical and family history, physical examination and imaging tests that showed enlarged vestibular aqueducts. Presence of CM in the presence of very high thresholds in the ABR click and the obtaining of thresholds for and in ABR tone pip 0.5 kHz may not be adequate to differentiate between SNHL and other conditions associated with AN	There is a need to review the NHS/AN protocol used in the UK and a new study to establish parameters to aid in the differential diagnosis of CM. A holistic and audiological medical approach is essential to manage infants who fail at the NHS
Riazi and Ferraro	2008/USA	Case reports	C4	To evaluate techniques that can optimize the recording of CM in humans. Through a variety of stimulus parameters and shielding conditions aimed at inhibiting/reducing artifacts that can contaminate the CM	11	7 children and 4 adults	Not reported	TOAE, Ecog, ABR, by click and toneburst (500, 1000 and 2000 Hz)	The results suggest that it is easier to separate the CM of the artifact from the stimulus using an electrode in the auditory canal and toneburst stimuli. Additionally, electromagnetic shielding and grounding of the power cables and the acoustic transducer were effective in reducing and/or eliminating the stimulus artifact	The results of this normative study may help improve the diagnosis of CM in AN and other hearing-related disorders
Talaat et al.	2009/Egypt	Prevalence Study	B2B	Detect the prevalence of AN in children and young individuals with severe to profound hearing loss	112	6–32 months	19 months	Behavioral audiometry or Visual Boost, ABR by click and Toneburst (500 Hz), acoustic immittance test	15 patients were diagnosed according to our diagnostic criteria	The prevalence of AN in the study group was 13.4%. We recommend the CM recording to be routinely tested during the evaluation of ABR whenever the results obtained are altered
Mo et al.	2010/China	Observational Cross-sectional	C4	Describe the audiological findings of AN	48	6–58 months	Not reported	Behavioral audiometry, DPOAE, ABR by click and acoustic immittance test	There were 40 children with a bilateral AN profile and 8 unilateral cases; in the contralateral ears of these cases, there were 3 ears with ABR thresholds that were better than 30 dB NHL, which indicates normal auditory function, and 5 with absent or severely altered ABR. In addition, four children were diagnosed with Auditory Nerve Disabilities after further investigation through inner ear magnetic resonance imaging	The audiological results in this group of children show variability in relation to the ABR thresholds and the wave morphology, the behavioral thresholds and presence of CM and DPOAE. This may reflect the heterogeneous nature of the AN. Additionally, concomitant pathologies of the inner ear or from the middle ear disorders may disclose AN. Absent or severely altered ABR together with the presence of CM are the most reliable measures to detect AN
Shi et al.	2012/China	Observational Cross-sectional	C4	Investigate the characteristics and clinical significance of CM in the diagnosis of AN in infants and children	36	3 months to 9 years	3 years	Behavioral audiometry, DPOAE, ABR by click and acoustic immittance testing	There was no significant difference in the length or amplitude of CM between the group with AN and the group with normal hearing. But the amplitudes of the CM with AN and absent DPOAE were significantly lower than in individuals with normal hearing	The CM may be very important in the diagnosis of AN. The maximum amplitudes of the CM were always found at around 0.6 ms. It is more useful for the diagnosis of AN to analyze the patterns of CM amplitudes and functions of OHC and IHC together
Liu et al.	2012/China	Retrospective cross-sectional cohort	C4	Explore a possible correlation between cochlear nerve impairment and unilateral AN	85	2–23 years	Not reported	Pure tone audiometry, DPOAE, TOAE, ABR by click and acoustic immittance testing	Eight cases were diagnosed with unilateral AN caused by cochlear nerve impairment. 7 had a type “A” tympanogram with normal bilateral OAE; the last one had unilateral type “B”, tympanogram, absent OAE and present CM, according to alterations in the middle ear. ABR was absent in all patients and neuronal responses from the cochlea were not disclosed when viewed by magnetic resonance imaging of the internal auditory canal	The cochlear nerve impairment can be seen by electrophysiological evidence and may be an important cause of unilateral AN. Magnetic resonance imaging of the internal auditory canal is recommended for the diagnosis of this disease
Penido and Issac	2013/Brazil	Cohort study	C4	Determine the prevalence of AN in individuals with SNHL	2292	0–95 years	Not reported	Pure tone and vocal audiometry; acoustic immittance testing; OAE; ABR and CM	1.2% had AN. Of these, 29.6% had mild SNHL; 55.5% moderate; 7.4% severe and 7.5% profound. 14.8% were aged 0–20 years; 33.4% were 21–40 years; 44.4% were 41–60 years and 7.4% were older than 60 years	The prevalence of AN was 1.2% in individuals with SNHL

ABR, auditory brainstem response; OAE, otoacoustic emissions; TOAE, Transient otoacoustic emissions; DPOAE distortion product otoacoustic emissions; CM, cochlear microphonism; AN, Auditory Neuropathy; AD, auditory dyssynchrony; NHS, Neonatal Hearing Screening; Ecog, Electrocochleography; EcogT, tympanic electrocochleography; EcogET, Extratympanic Electrocochleography; SNHL, Sensorineural Hearing Loss; OHC, outer hair cells; IHC, inner hair cells; CAP, Composite Action Potential.

## Discussion

Due to the recent increase in the number of studies on AN, this review shows that most studies were published between 1996 and 2014. All selected articles associated AN with the MC recording through two specific tests, ABR and the Ecog, using invasive or non-invasive methods, in addition to other tests to assess auditory function.

There was greater investment in research in this area in the late 90s, when AN was described.^1^ Since then, studies have sought to explain the location of the lesion, risk factors, prevalence and more accurate diagnostic tests in AN.

Regarding location, the literature indicates a broad possibility, as the lesion may occur in several structures or in more than one at the same time, such as the IHC, auditory nerve fibers, or in their synapses.^9^ Another study suggests that there is an abnormality in the auditory system, located in the VIII nerve, ganglion neurons, in the IHC, between their synapses or a combination of them.^1^

Risk factors are usually associated to neonatal problems such as prematurity, low birth weight, anoxia, hypoxia, hyperbilirubinemia, need for mechanical ventilation and intracranial hemorrhage,^10^ as well as genetic and mitochondrial disorders^11^ and a family history of hearing disorders.^3^, ^12^

According to the studies shown in this review, the prevalence of AN in children and young individuals with severe to profound hearing loss was 13.4%^9^ and 1.2% in individuals with SNHL.^13^ The prevalence has also been described in children with risk criteria for AN as 1 in 433 (0.23%) and in the group of children with permanent hearing deficit, it was 1 in 9 (11.01%).^5^ Another study indicates a prevalence of 8.44% in a group of 379 children with ABR alteration.^4^

There is an agreement in the reviewed literature regarding examination findings in patients with neuropathy, who have present OAE and CM, absent or very altered ABR and absent acoustical reflexes.^14^, ^9^, ^10^, ^15^, ^12^, ^16^, ^17^, ^18^, ^19^, ^20^, ^21^, ^13^, ^22^, ^23^ In audiometry, the described pattern is permanent or fluctuating hearing loss of varying degrees, with flat or ascending audiometric configurations,^12^, ^17^ in addition to difficulties in speech perception, especially in the presence of noise.^14^, ^9^, ^10^, ^15^, ^12^, ^16^, ^17^, ^22^, ^23^ The OAE are present, but they may disappear with time.^16^ The results of objective electrophysiological tests such as presence of TOAE, absent or very altered ABR and presence of CM have emerged as the first diagnostic tool for AN in infants.^4^, ^24^ Additionally, patients with AN have an alteration in OAE suppression effect caused by the efferent auditory pathways.^25^ The absence of OAE suppression suggests that the olivocochlear efferent function is altered.^24^

Considering the findings of the auditory function tests, the presence of CM becomes the determinant finding in the differential diagnosis of AN.^16^

The protocol used to record the CM by ECoG or ABR should always reverse the stimulus polarities to confirm the recording inversion and, therefore, confirm CM.^16^, ^17^, ^19^, ^13^, ^22^ Furthermore, the use of insert earphones is important to allow the blocking of the plastic tube, indispensable to confirm the biological response, discarding the presence of electrical signal artifact.^17^, ^20^, ^13^ Insert earphones should always be used in the ABR to allow stimulus artifacts to be separated from cochlear potentials.^2^ Another study also confirmed the CM response by closing the stimulus tube to prevent the acoustic signal from reaching the ear canal, eliminating the artifacts.^5^

Some studies have reported the use of Ecog as a diagnostic test for AN. But there are reports suggesting that the Transtympanic Electrocochleography (EcogT) is the gold standard tool to evaluate CM,^9^, ^16^, ^17^ because Ecog allows a more detailed analysis of cochlear function in relation to ABR.^9^, ^17^ However, promontory recordings are considered more sensitive than the ear canal and that results in a better signal-to-noise ratio, as the CM comes first from the basal portions of the cochlea, with a negligible contribution of the apical regions.^7^

In one of the reviewed studies, no significant difference was found between the amplitude of the CM in normal hearing individuals and those with AN. The maximum amplitudes of CM for almost all patients were around 0.6 ms after the stimulus.^13^ The literature reports that CM in patients with AN are especially prominent and persist for several milliseconds after a transient stimulus.^2^, ^24^ Another study reported that the mean amplitudes of the CM was 0.4 ms in patients with AN, significantly higher than in individuals with normal hearing.^24^

The duration of the CM was longer in the group with AN than in the group with normal hearing.^16^, ^13^ In patients with AN in the ABR, the CM appears wide and can exhibit a duration of up to 4–6 ms, and may be mistaken for electrical activity of the brain stem; however it does not change with decreasing intensity, but with the reversed stimulus polarity.^25^

In general, the assessed literature agrees on the location, risk factors and clinical findings of AN and reports that its differential diagnosis is confirmed based on the CM recording, because even at an advanced state of AN, CM remains present.

This review includes studies that describe the tests most commonly used to describe the characteristics of Cochlear Microphonism in Auditory Neuropathy/Dyssynchrony. For that purpose, several types of studies were selected, which may seem like a limitation, but on the other hand, they may have different perspectives on the subject, always taking into account the previously defined selection criteria.

## Conclusion

Based on the studies included in this literature review, we conclude that:

The presence of the CM is a crucial finding in the differential diagnosis of AN.
The CM recording protocol must include the use of insert earphones and reverse polarity and the stimulus blocking to prevent electrical artifact interference.
The amplitude of CM in AN showed no significant difference when compared with the amplitude of CM in individuals with normal hearing.
The duration of CM is longer in individuals with AN.

## Conflicts of interest

The authors declare no conflicts of interest.
